# Population analysis of *Vibrio parahaemolyticus* originating from different geographical regions demonstrates a high genetic diversity

**DOI:** 10.1186/1471-2180-14-59

**Published:** 2014-03-08

**Authors:** Sara Urmersbach, Thomas Alter, Madura Sanjeevani Gonsal Koralage, Lisa Sperling, Gunnar Gerdts, Ute Messelhäusser, Stephan Huehn

**Affiliations:** 1 Institute of Food Hygiene, Freie Universitaet Berlin, Koenigsweg 69, 14163 Berlin, Germany; 2 Government Veterinary Office, Walikanda 51070, Polonnaruwa, Sri Lanka; 3 Alfred Wegener Institute for Polar and Marine Research, 27498 Heligoland, Germany; 4 Bavarian Health and Food Safety Authority, Oberschleißheim, Germany

**Keywords:** MLST, Multilocus sequence typing, *Vibrio parahaemolyticus*, Population structure

## Abstract

**Background:**

*Vibrio parahaemolyticus* is frequently isolated from environmental and seafood samples and associated with gastroenteritis outbreakes in American, European, Asian and African countries. To distinguish between different lineages of *V. parahaemolyticus* various genotyping techniques have been used, incl. multilocus sequence typing (MLST). Even though some studies have already applied MLST analysis to characterize *V. parahaemolyticus* strain sets, these studies have been restricted to specific geographical areas (e.g. U.S. coast, Thailand and Peru), have focused exclusively on pandemic or non-pandemic pathogenic isolates or have been based on a limited strain number.

**Results:**

To generate a global picture of *V. parahaemolyticus* genotype distribution, a collection of 130 environmental and seafood related *V. parahaemolyticus* isolates of different geographical origins (Sri Lanka, Ecuador, North Sea and Baltic Sea as well as German retail) was subjected to MLST analysis after modification of *gyr*B and *rec*A PCRs. The *V. parahaemolyticus* population was composed of 82 unique Sequence Types (STs), of which 68 (82.9%) were new to the pubMLST database. After translating the in-frame nucleotide sequences into amino acid sequences, less diversity was detectable: a total of 31 different peptide Sequence Types (pSTs) with 19 (61.3%) new pSTs were generated from the analyzed isolates. Most STs did not show a global dissemination, but some were supra-regionally distributed and clusters of STs were dependent on geographical origin. On peptide level no general clustering of strains from specific geographical regions was observed, thereby the most common pSTs were found on all continents (Asia, South America and Europe) and rare pSTs were restricted to distinct countries or even geographical regions. One lineage of pSTs associated only with strains from North and Baltic Sea strains was identified.

**Conclusions:**

Our study reveals a high genetic diversity in the analyzed *V. parahaemolyticus* strain set as well as for geographical strain subsets, with a high proportion of newly discovered alleles and STs. Differences between the subsets were identified. Our data support the postulated population structure of *V. parahaemolyticus* which follows the ‘epidemic’ model of clonal expansion. Application of peptide based AA-MLST allowed the identification of reliable relationships between strains.

## Background

*Vibrio* (*V.*) *parahaemolyticus* is naturally present in coastal waters worldwide [1-4]. It is associated with self-limiting gastroenteritis due to the ingestion of contaminated raw or undercooked seafood [5,6]. In 1996 the pandemic O3:K6 serotype emerged in Asia and was identified as the predominant cause of numerous outbreaks throughout the world [7-10]. In recent years, other serotypes, esp. serovariants of O3:K6, were associated with severe outbreaks [10].

To distinguish between different lineages of *V. parahaemolyticus* various techniques have been used so far (e.g. serotyping, PFGE, rep-PCR), most promising multilocus sequence typing (MLST). In MLST analysis the genotypic relatedness of bacterial strains is analyzed basing on the sequences of internal fragments of usually 6 to 8 housekeeping genes [11,12]. Due to the nucleotide sequence based typing the comparison of results obtained by others and exchange via public databases is possible and allows continuously increasing understanding of the molecular epidemiology and evolution of the typed bacteria [12-14].

The population of *V. parahaemolyticus* is characterized by a high degree of genotypic diversity that diversifies in the first step via recombination and is thus called a semi-clonal population [13,15]. In its habitat the marine and estuarine environment *V. parahaemolyticus* encounters changing environmental conditions [4]. Better adapted or faster adapting clones arise from the background of the diverse and highly recombinogenic bacterial population leading to the “pandemic” model of clonal expansion [16]. In MLST analyses such adapted clones are termed clonal complexes (CC) and are characterized by strains of allelic profiles or Sequence Types that differ in at most one allele. The pandemic clone of *V. parahaemolyticus*, consisting of O3:K6 strains and its serovariants, shares the same genetic properties (*trh*^-^, *tdh*^+^, GS-PCR^+^) and forms the distinct cluster of clonal complex 3 (CC3) founded by Sequence Type 3 (ST3). On the contrary the converse argument is not true as CC3 is also formed by non-pathogenic strains [17]. Since ST and serotype are not linked, a diverse set of serotypes constitutes ST3 (largely caused by serotype switching via recombination) [9,13,17-20].

The overall genotypic diversities differ depending on the pathogenicity of strains: Pandemic strains show a high uniformity, whereas non-pandemic strains are highly diverse, leading to the observation that an analyzed geographically restricted subpopulation was genetically as diverse as the entire worldwide pubMLST database [21-24]. In contrast, environmental *tdh*^+^/*trh*^+^*V. parahaemolyticus* are as diverse as the non-pathogenic populations [25]. Diversity also depends on water temperature, with a less diverse cold water adapted population replaced by more diverse strains when temperature rises [23]. The environmental populations are characterized by a fast evolution observable in the rapid turnover of predominant strains [25,26]. But some clones and strain groups can persist for years in a specific habitat, creating an endemic population [23]. With the application of MLST a high degree of genetic similarity between environmental and pandemic or non-pandemic infectious isolates as well as the mentioned environmental clade of CC3 isolates was shown, emphasizing the potential threat even of environmental strains to human health [27]. A clustering of strains in regard to specific properties, like sampling time, habitat or origin is desired to establish a relationship between these properties and the genotype (in the case of MLST the ST) of a strain. However, in the case of *V. parahaemolyticus* this was not possible in general [13,19,25]. Theethakaew *et al*. were able to identify distinct clusters of strains sampled either from farmed prawns or clinical cases [24]. Due to the high genetic diversity especially of environmental strains, the identification of related strains can lack reliability; therefore clustering of strains on the basis of their amino acid sequence was applied to *V. parahaemolyticus*[24,28].

Even though some studies already used MLST analysis to characterize *V. parahaemolyticus* strain sets, they were restricted to specific geographical areas (e.g. U.S. coast, Thailand and Peru) [23,24,27,29], focused exclusively on pandemic or non-pandemic pathogenic isolates [17,21,22,25,26,29] or were based on a limited strain number. Whether strains, originating from a specific habitat but of different geographic regions, possess similar properties concerning their genetic diversity has not been investigated yet.

Thus, the goal of this study was to investigate four different *V. parahaemolyticus* strain sets, each of distinct geographical origin (a cold water population originating from the German North Sea and the Baltic Sea, two prawn associated strain sets originating from Sri Lanka and Ecuador and additionally seafood isolates from German retail) by using MLST analysis, in order to define sequence polymorphism of the strains, investigate genetic polymorphisms and relationships among strains of the different regions and to analyze the probable evolutionary relationships among the strains. Therefore differences in the relationship of isolates in regard to sequence type, clonal complex and peptide sequence type affiliation were considered. To analyze peptide based differences a peptide-based MLST scheme was implemented into the pubMLST database. To obtain a more global overview previously available MLST data of isolates from other countries and continents were included.

## Methods

### Sampling of *Vibrio parahaemolyticus* isolates

A total of 130 *V. parahaemolyticus* isolates from different geographical areas were analyzed. The strain set consisted of four groups based on the geographic origin of strains and the sampling events: the first group was obtained from prawn farms located in three Sri Lankan regions (n = 43) [30], the second group consists of strains (n = 34) that were isolated from regional and imported food samples in Germany (at retail) of different geographic origins and sample types. Within the third group 27 isolates obtained from local markets and prawn farms in Ecuador are grouped. Finally the fourth group consists of planktonic isolates from the North Sea, the Kattegat, the Skagerrak and the Baltic Sea (NB-Seas; n = 26). Additionally, the two Japanese clinical strains *V. parahaemolyticus* ATCC 17802 and RIMD 2210633 served as reference strains for process control. Details on the individual strains are summarized in Additional file 1: Table S1. Rarefaction curves for the whole strain set, for the three geographical subsets as well as for the entire pubMLST dataset were calculated to evaluate if sampling was adequate and if the existing diversity was recorded [31].

Isolates were stored in Cryovials at –80°C (Cryobank; Mast Diagnostica, Bootle, UK).

### MLST analysis

Prior to DNA analysis strains were grown overnight in alkaline peptone water (APW; 0.3% yeast extract, 1% peptone, 2% NaCl, pH 8; Merck, Darmstadt, Germany) at 37°C with shaking (200 rpm). Bacterial DNA was extracted using Chelex 100 Resin (BioRad, Hercules, USA) according to the manufacturer’s instructions.

For MLST analysis, internal fragments of the genes *dna*E, *gyr*B, *rec*A, *dtd*S, *pnt*A, *pyr*C and *tna*A were amplified by PCR and sequenced using primers and protocols described on the *V. parahaemolyticus* MLST website [13,14,32]. Sequencing was performed in both directions. Sequences were edited and the complementary fragments of each locus of the individual isolate were assembled, trimmed and aligned in Bionumerics v 6.01 (Applied Maths, Sint-Martens-Latem, Belgium). The consensus sequences were queried against the pubMLST database to determine the allele designations and Sequence Type (ST) of each isolate. Sequences of new alleles and new allelic profiles were submitted to the pubMLST database and were assigned new numerical identifiers.

As observed by others, amplification and sequencing of *gyr*B and *rec*A with the original primers has not always led to results [17]. Therefore, each of these genes was divided into two fragments (*gyr*B-up, *gyr*B-down, *rec*A-up, and *rec*A-down). Two inner primers were designed (*gyr*B-up_rev: [M13-rev]CGATTCAACCGCTGATTTCACTTC; *gyr*B-down_for: [M13-for]GCGGCACTAACACGTACGCTAAAC; *rec*A-up_rev: [M13-rev]ACGGATTTGGTTGATGAAGATACA; *rec*A-down_for: [M13-rev]GGGTCTCCAAGCTCGTATGC) and ‘5′-tailed’ with the universal M13 primers (M13-for: TGTAAAACGACGGCCAGT and M13-rev: CAGGAAACAGCTATGACC). This enabled PCR amplification and sequencing with the conditions and in combination with the original primers published by González-Escalona *et al.*[13].

### Peptide sequence type designation

Translating the in-frame nucleotide sequences into the peptide sequences allows an analysis on the phenotypic level, as only non-synonymous substitutions of nucleotides leading to a different amino acid were considered. Similar to the nucleotide sequences, each unique peptide sequence was assigned a distinct numerical identifier and the different combinations of alleles at each locus lead to the allelic profile at peptide level. Each individual profile was transformed to a peptide Sequence Type (pST) that allows the unambiguous identification of a clone. The peptide sequences and peptide profiles of the entire pubMLST dataset were submitted to the pubMLST database and implemented as an additional typing scheme, called AA-MLST, accessible at the pubMLST web page [32]. The loci were labeled with the prefix ‘p_’ and the appropriate locus designation.

### Data analysis

#### Phylogenetic analysis

The generated sequence data were analyzed using Bionumerics and compared to already accessible sequences on the pubMLST web page [32]. To visualize the clonal relationship between isolates of subsets and in context with the entire dataset stored in the pubMLST database the goeBURST algorithm was used [33,34]. By using the allelic profile data - on nucleotide and peptide level, respectively - isolates were subdivided into groups of related genotypes. Isolates that shared 100% identity in 6 of the 7 loci with at least one other member of the group, the single locus variants (SLVs), were assigned to a single clonal complex (CC). The algorithm also predicted the presumable founder (p)ST of each CC and any single and double locus variants originating. The algorithm was also used to obtain a ‘population snapshot’ with the group definition 0 of 7 loci shared and to create a fullMST, where all STs were connected [34,35].

UPGMA based on pairwise comparisons between the in-frame concatenated sequences were constructed (Bionumerics). Clusters were assigned for strains with more than 99% or 99.95% similarity for nucleotide and peptide data, respectively.

#### Population genetic analysis

The in-frame sequences at the seven loci were concatenated, leading to a sequence of 3669 bp in length for each strain. The numbers of polymorphic sites as well as the *d*_*N*_*/d*_*S*_ were calculated. The *d*_*N*_*/d*_*S*_-value was calculated by the Nei and Gojobori method as implemented in START2 [36,37]. The Simpsons Index of diversity (*D*) was calculated using Phyloviz to determine the discriminative ability of the different loci [33].

The population structure of *V. parahaemolyticus* was accessed by calculating the standardized Index of Association ($I_{A}^{S}$) implemented in START2 [37]. The calculation was applied to different sets of STs as performed by others [13,15,24].

## Results

### Diversity of strain collection

To evaluate completeness of the sampled diversity of strains present in the different geographical regions rarefaction curves were performed on the three geographical subsets, the complete strain set as well as on the entire pubMLST dataset. All rarefaction curves did not reach the plateau phase, indicating that some diversity remained unsampled (data not shown). Only the curve of Sri Lankan STs did approximate the plateau.

### Genotypic strain diversity and population genetic analysis

Summarized data on allelic profiles on nucleotide and peptide level and (p)STs of the analyzed strains along with strain information is presented Additional file 1: Table S1. The data on nucleotide and allelic diversity of the MLST and AA-MLST scheme are summarized in Table 1. All observations regarding the diversity of (p)STs, alleles, polymorphic sites, *d*_*N*_*/d*_*S*_ and *D* were in concordance to the obtained values calculated on basis of all pubMLST entries (Table 1).

**Table 1:** Properties and diversities of MLST and AA-MLST loci

**Locus**	**Fragment size**^**A**^		**Number and proportion of alleles**^**B**^		**Number and proportion of new alleles**		**Number and proportion of variable sites**^**B**^		***D *** **Simpsons Index of diversity**^**B**^		***d***_***N***_**/*d***_***S ***_**ratio**^**B**^^**C**^
	**MLST**	**AA-MLST**	**MLST**	**AA-MLST**	**MLST**	**AA-MLST**	**MLST**	**AA-MLST**	**MLST**	**AA-MLST**	**MLST**
*dna*E	555 bp	185 aa	55; 14.8% (195; 13.7%)	5; 12.8% (15; 10.6%)	13; 23.6%	2; 40.0%	55; 9.9% (115; 20.7%)	3; 1.6% (11; 5.9%)	0.988 (0.985)	0.630 (0.614)	0.026 (0.025)
*gyr*B	591 bp	197 aa	65; 17.5% (274; 19.2%)	1; 2.6% (7; 4.9%)	28; 43.1%	0; 0.0%	47; 8.0% (100; 16.9%)	*; - (6; 3.0%)	0.992 (0.989)	0.000 (0.094)	0.000 (0.002)
*rec*A	726 bp	242 aa	57; 15.3% (201; 14.1%)	1; 2.6% (9; 6.3%)	21; 36.8%	0; 0.0%	66; 9.1% (216; 29.8%)	*; - (24; 9.9%)	0.987 (0.985)	0.000 (0.106)	0.006 (0.015)
*dtd*S	456 bp	152 aa	55; 14.8% (237; 16.6%)	3; 7.7% (9; 6.3%)	17; 36.4%	1; 33.3%	50; 11.0% (100; 21.9%)	2; 1.3% (8; 5.3%)	0.983 (0.987)	0.127 (0.117)	0.002 (0.002)
*pnt*A	429 bp	143 aa	41; 11.0% (146; 10.3%)	7; 17.9% (36; 25.4%)	11; 26.8%	4; 57.1%	41; 9.6% (85; 19.8%)	6; 4.2% (29; 20.8%)	0.965 (0.966)	0.404 (0.525)	0.018 (0.042)
*pyr*C	489 bp	163 aa	55; 14.8% (219; 15.4%)	14; 35.9% (41; 28.9%)	20; 38.2%	3; 21.4%	48; 9.8% (107; 21.8%)	11; 6.8% (31; 19.0%)	0.986 (0.981)	0.791 (0.753)	0.045 (0.049)
*tna*A	423 bp	141 aa	44; 11.8% (152; 10.7%)	8; 20.5% (25; 17.6%)	15; 34.1%	4; 50.0%	41; 9.7% (89; 21.0%)	6; 4.3% (22; 15.6%)	0.974 (0.97)	0.355 (0.440)	0.019 (0.023)
total	3669 bp	1223 aa	372; 100% (1424; 100%)	39; 100% (142; 100%)	125; 33.6%	15; 29.9%	348; 9.5% (812; 22.1%)	28; 2.3% (131; 10.7%)			

^A^in-frame fragment size.

^B^Values in parentheses are computed based on the data of the entire pubMLST dataset.

^C^*d*_*N*_/*d*_*S*_ ratio, ratio of non-synonymous to synonymous mutations, value <1 indicates purifying selection of loci.

*For the one occurring allele no variable sites could be determined.

#### Diversity of sequence types

By applying MLST analysis, the 130 strains analyzed in our study resulted in 82 unique STs of which 68 (82.9%) were new in comparison to pubMLST database entries. Even after dividing the total collection into geographical subsets, the number of different and new STs remained high (Table 2). Individual STs were mostly recovered once, but (especially for the Sri Lankan strains) specific STs (e.g. STs 394, 395, 397) were more frequently isolated, thereby the most frequently identified ST was ST394 (7.7%) and the 64 least frequent STs occurred only once (each 0.8%) (Additional file 1: Table S1).

**Table 2:** **Properties of the analyzed *V*. *parahaemolyticus *** **populations**

	**Number of isolates**	**Number of STs (new STs)**		**Number of pSTs (new pSTs)**	
Sri Lanka	43	16	(15)	9	(4)
-Chillaw	11	6	(6)	6	(1)
-Puttalam	21	12	(11)	7	(3)
-Madurankuliya	11	6	(5)	4	(1)
Ecuador	27	21	(19)	13	(8)
-market	9	8	(8)	6	(4)
-Machala	10	8	(6)	6	(2)
-Balao	2	2	(2)	2	(1)
-Guayaquil	6	5	(5)	3	(1)
NB-Seas	26	19	(16)	13	(6)
-North Sea	8	4	(4)	4	(2)
-Baltic Sea	14	11	(8)	8	(2)
-Kattegat and Skagerrak	4	4	(4)	4	(2)
German retail	34	29	(21)	10	(3)
All isolates	130	82	(68)	31	(19)

The individual loci possessed 41 (*pnt*A) to 65 (*gyr*B) unique alleles of which 23.6% (*dna*E) to 43.1% (*gyr*B) were new, leading to a total of 125 (33.6%) alleles new to the database. Up to 40.9% of the individual alleles at a single locus were present in more than one distinct ST.

The distinct alleles were characterized by different numbers of variable sites with *gyr*B as the most diverse locus possessing only 47 (8%) variable sites. The higher number of combinations of different SNPs led to the high number of distinct alleles. The *d*_*N*_/*d*_*S*_ value indicates the kind of selection in a chosen gene and population: a *d*_*N*_*/d*_*S*_ < 1 is indicative of purifying selection, *d*_*N*_*/d*_*S*_ = 1 of neutral selection and *d*_*N*_*/d*_*S*_ > 1 of positive selection. The *d*_*N*_/*d*_*S*_ values for all loci were zero or close to zero.

The Simpsons Index of diversity (*D*) measures the ability of a typing scheme to distinguish between unrelated strains [38]. This value indicates for all MLST loci a high ability to differentiate strains with *pnt*A (0.965) being the least and *gyr*B (0.992) the most differentiating (Table 1).

#### Diversity of peptide sequence types

After translating the in-frame nucleotide sequences into the peptide sequences a total of 31 different pSTs with 19 (61.3%) new pSTs were generated from the analyzed isolates (Additional file 1: Table S1). The pSTs occurred with a frequency of 0.8% to 28.5%. For the different loci a total of 39 distinct alleles were found. For most of the loci, one allele was dominant (more than 90%), except for p_dnaE and p_pyrC. New alleles (n = 15) were identified for all loci despite of p_gyrB and p_recA. The Simpsons Index of diversity was heterogenic, with very low values for p_gyrB, p_recA and p_tnaA (0.000, 0.000, and 0.127) indicating a low ability to discriminate between strains up to higher values for p_dnaE and p_pyrC (0.630 and 0.791) (Table 1).

To summarize the data of the different subpopulations, less different pSTs with a lower proportion of new types were observed, but for several regions pSTs were diverse, e.g. each distinct ST of strains from the Chillaw region in Sri Lanka possessed a unique corresponding pST (Table 2).

#### Peptide sequence types of pubMLST database

In total, 584 STs with at least one corresponding isolate were present in the pubMLST database and translation of the in-frame sequences yielded 166 distinct pSTs. AA-MLST profiles and properties of each allele on peptide level (numbers, sequences and frequencies) are shown in Additional file 2: Tables S2. An alternative AA-MLST typing scheme was applied by Theethakaew *et al*. during the preparation of this manuscript [24].

#### Comparison of MLST and AA-MLST

In total, 372 unique MLST and 39 AA-MLST-alleles were detected in our study. Therefore most of the reduction (mean of 95.6%) in strain diversity stemmed from the wobble bases as exemplarily calculated for the most common allele of each locus of the pubMLST dataset (data not shown). The proportion of the alleles of one locus to the total number of alleles changed from nucleotide to peptide level as reflected by the *d*_*N*_*/d*_*S*_-values and revealing different influences of the loci on both typing schemes. For example, on nucleotide level 65 different *gyr*B alleles were transformed into one p_gyrB. This is reflected by a *d*_*N*_*/d*_*S*_-value of 0 that indicates exclusively synonymous substitutions. In contrast, far more non-synonymous substitutions (as indicated by a *d*_*N*_*/d*_*S*_-value of 0.045) were observed for *pyr*C.

### Clonal relationships among global sets and subsets of isolates

To identify the population structure of the analyzed strains, the standardized Index of Association ($I_{A}^{S}$) was calculated (Table 3). The value differed significantly from zero, when all our isolates, all subsets separately or all pubMLST isolates were included, indicating that the alleles were in linkage disequilibrium or were not randomly distributed. When analyzing only one isolate per ST, the $I_{A}^{S}$ drops, but remains unequal to zero, indicating a tendency to linkage disequilibrium.

**Table 3:** **Standardized Index of Association of different *V*. *parahaemolyticus *** **populations to assess population structure**

	**Number of isolates**	**Standardized index of association**$I_{A}^{S}$
Sri Lankan isolates	43	0.8043 (sld)
Ecuadorian isolates	30	0.6277 (sld)
Isolates from NB-Seas	36	0.6482 (sld)
All isolates from this study	130	0.4922 (sld)
pubMLST isolates	1089	0.6291 (sld)
One isolate per ST	584	0.0841 (sld)

(sld) significant linkage disequilibrium.

#### Global analysis

**Figure 1:**
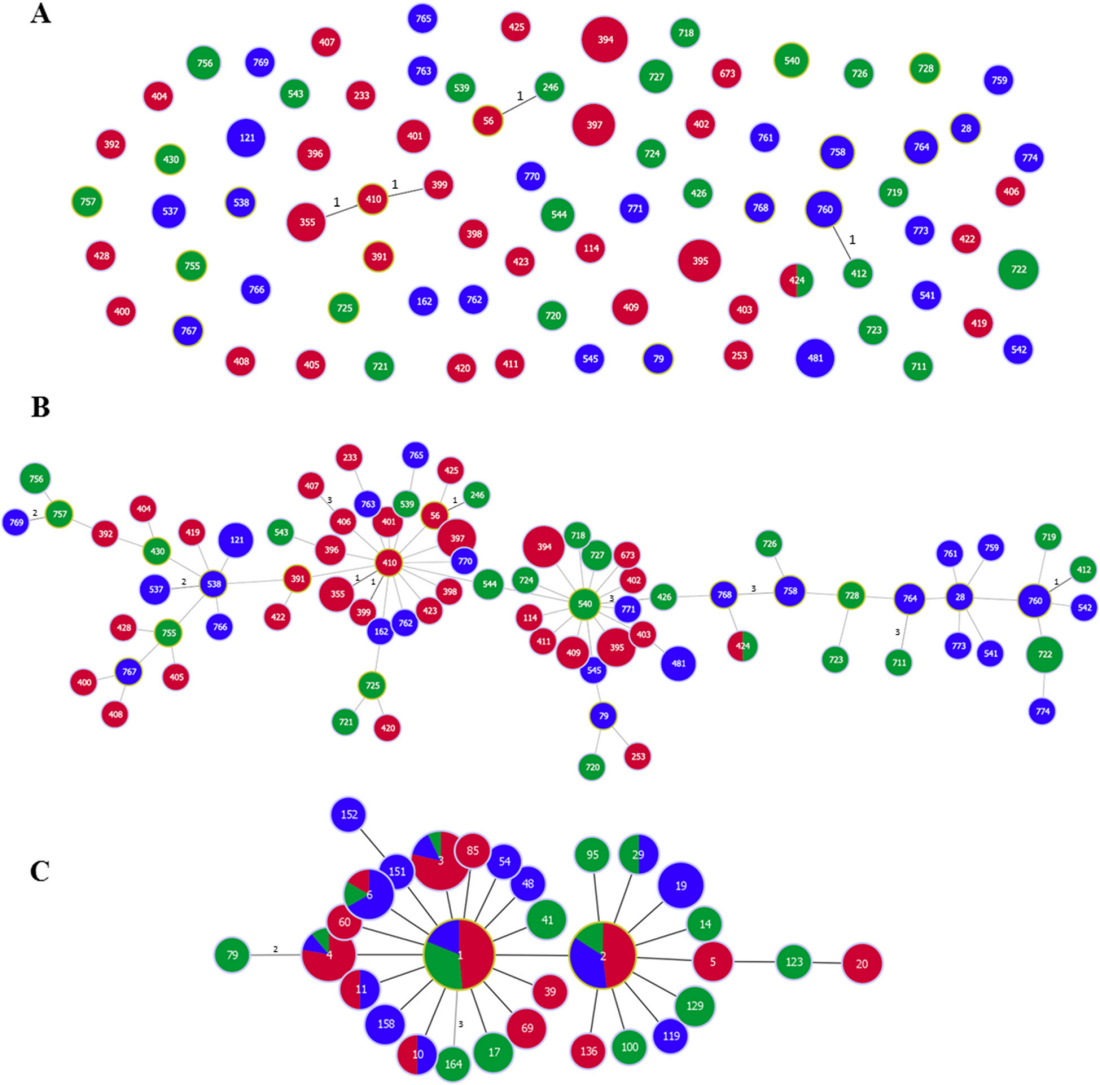
**MSTs based on allelic profiles.** Coloring depends on geographical origin of isolates: Asia (red), South America (green), and Europe (blue). Size of circles represents number of isolates with the corresponding ST or pST. Circles surrounded by a light green circle were (sub-) group founders. **A** Population snapshot based on MLST profiles. STs that differ in one allele are connected via black lines. **B** FullMST based on MLST profiles. The number of different alleles is indicated in the case of SLVs, DLVs and TLVs. All connections were drawn. SLVs are connected via black, DLVs via dark grey, TLVs via grey and all connection with a higher level via light grey lines. **C** FullMST based on AA-MLST profiles. The number of different alleles is indicated in the case of DLVs and TLVs all other pSTs are SLVs.

To gain an overview of clonal relations within the analyzed strains, a ‘population snapshot’ was obtained via goeBURST analyses (Figure 1A). The strains were assigned to one triplet (ST355-ST410-ST399) and two doublets (ST246-ST56 and ST760-ST412). The remaining 75 STs were singletons. When including double locus variants (DLVs) and triple locus variants (TLVs) as well 6 more doublets were identified (Figure 1B). For these groups, the strains were either isolated from one continent or two, demonstrating the possibility for a global dissemination of CCs. When the level is increased to seven, all STs were connected (Figure 1B).

To show clonal relationships, an AA-MLST scheme was implemented. When analyzing a ‘population snapshot’ on peptide level, only pST79 and pST164 differed in more than one allele to all other pSTs, leading to a single complex founded by pST1 and pST2 (Figure 1C). Thus the genotypic relatedness was more reliable on peptide level than on nucleotide level.

No general clustering of strains from specific geographical regions was observed. The most common pSTs were found on all continents. Nonetheless, one lineage of specific pSTs was identified: pST151 and pST152 exclusively occurred in strains isolated from NB-Seas (Figure 1C).

By analyzing our strains in combination with all pubMLST strains, 3 CCs, 6 triplets and 10 doublets contained STs from this study (Additional file 3: Figure S1). Formation of a new CC (with the founder ST412) was observed. ST412 was identified in a prawn associated Ecuadorian strain, whereas three STs of the same CC belonged to potentially pathogenic environmental U.S. strains (ST313, ST314 and ST315) and one ST (ST760), carried by one of our strains isolated from German North Sea waters. By including also DLVs, two STs were assigned to this CC that originated from environmental and clinical (ST43) or exclusively clinical (ST44) U.S. strains.

In the corresponding fullMST (Additional file 4: Figure S2) no clear groups were visible. Since the database consists of approx. 60% Asian isolates, a bias towards this region is obvious. Altogether, the reliability of this fullMST is partly weak: many connections are drawn on third or higher level, although they were connecting groups of strains with reliable relationships, as they are SLVs or DLVs. On peptide level (Additional file 5: Figure S3) no clear groups were visible. Nonetheless, lineages could be identified, that contained predominantly pSTs recovered from strains that originated from one continent (e.g. pST120-pST121-pST122 with Asian pSTs) and lineages that contained less Asian pSTs compared to other lineages (e.g. pST3, pST6 and pST8 with their descendants). The pSTs that were common within our strain collection were also the most common pSTs in the pubMLST dataset (e.g. pST1, pST2, pST3 and pST4).

#### Geographical subsets

**Figure 2:**
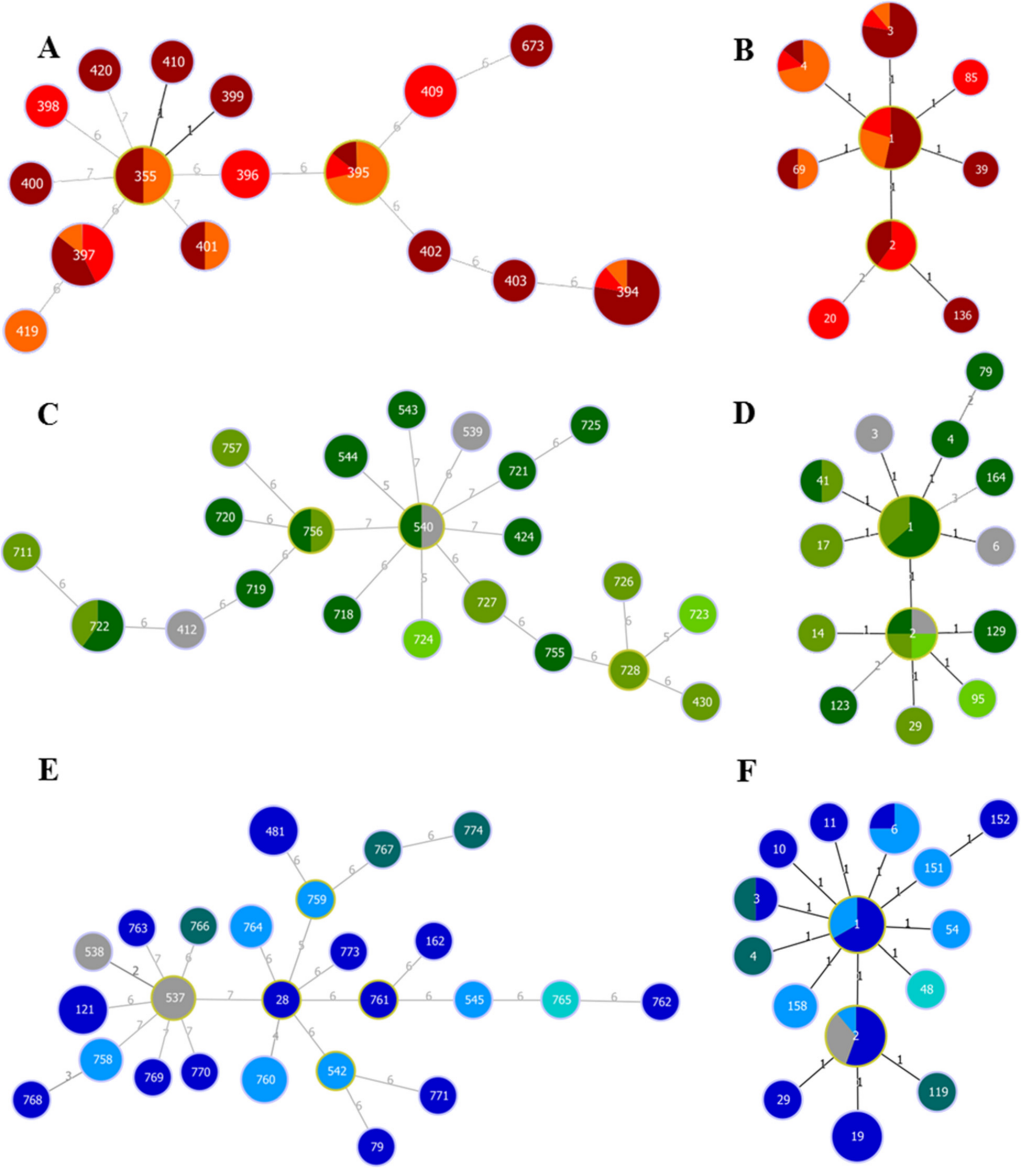
**FullMST of geographical subsets: A**, **C**, **E** based on MLST profiles and **B**, **D**, **F** based on AA-MLST profiles. **A** and **B** Sri Lankan subset (Puttalam-dark red, Chillaw-red, Madurankuliya-light red), **C** and **D** Ecuadorian subset (Machala-dark green, Guayaquil-green, Balao-light green), **E** and **F** NB-Seas subset (Baltic Sea-dark blue, North Sea-light blue, Kattegat-dark turquoise, Skagerrak-light turquoise). For all subsets: grey circles indicate STs whose regional origin is unknown. Black lines connect SLVs, dark grey lines connect DLVs and grey lines connect TLVs and light grey lines connect connections on higher level. Circles circled by a light green line were (sub-) group founders.

Figure 2 shows the regional distribution of strains (based on MLST data and AA-MLST data) within individual geographical regions (Sri Lanka, Ecuador or NB-Seas). The only identified triplet was formed by three Sri Lankan STs (Figure 2A). For the other subsets no SLVs were identified. Among the STs that were recovered more than once were either STs present in exclusively one region, as most of the Ecuadorian and NB-Seas STs (e.g. ST760, ST758, ST727), or STs that were distributed in more than one region, especially in Sri Lanka (e.g. ST394, ST395, ST397). There was no predominant ST that either dominated the subsets or was found in all of the geographical subsets. No ST was recovered in more than one subset (except ST424 in Sri Lanka and Ecuador), thus most of the STs did not show a global dissemination.

Common pSTs (low numbered pSTs like pST1to pST4) were found in all three subsets, two of the less common pSTs (pST6 and pST29) were found in Ecuador and NB-Seas, whereas the majority of the rare pSTs were exclusively found in one region. For the Sri Lankan subsets, a higher proportion of pSTs was present in more than one region, than for the Ecuadorian and NB-Seas subsets (Figures 2B, D and F). For the NB-Seas subset, STs that occurred multiple times were either recovered from North Sea isolates (ST758, ST760 and ST764) or Baltic Sea isolates (ST481) but not from both (Figure 2E).

Two STs were found in the retail samples too: ST394 was found in a sample taken from Sri Lankan prawn farms and in a German retail sample originating from the Indian Ocean; ST540 isolates were recovered from Ecuadorian prawn farms as well as from a German retail sample originating from Ecuador.

### Phylogenetic analysis

#### Global dataset

**Figure 3:**
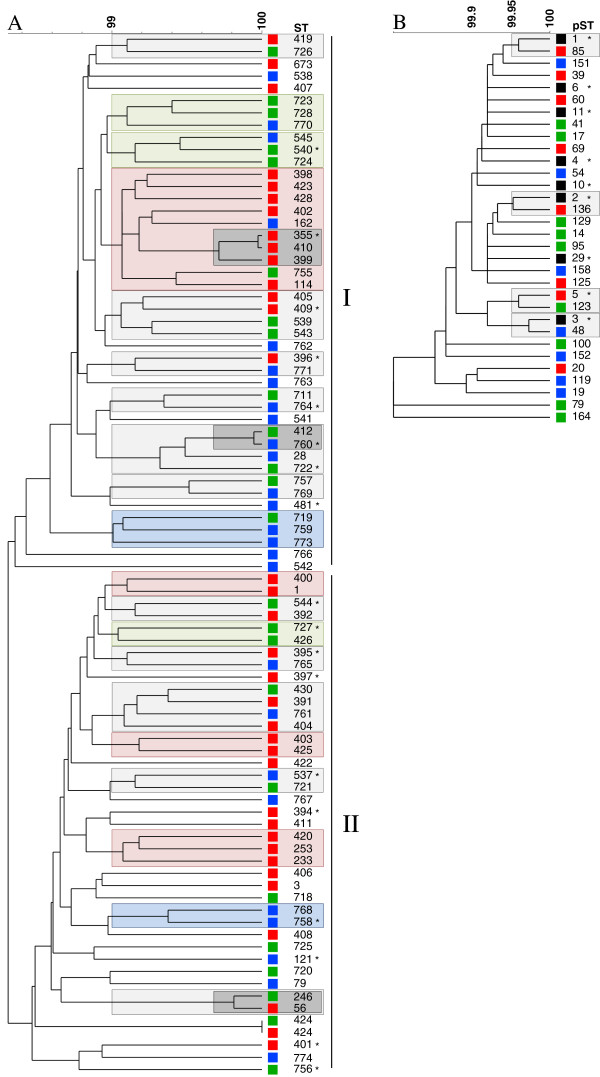
**UPGMA tree constructed from the concatenated nucleotide (A) and peptide (B) sequences of 130 isolates.** Squares next to the tree are colored regarding geographical origin (Asia-red, South America-green, Europe-blue and diverse origin-black). **A** STs of strains are shown and asterisks (*) mark STs found in more than one isolate originating from the same continent. Boxes mark cluster of isolates with more than 99% similarity. Coloring of boxes indicates the origin of majority of strains, while light grey boxes are indicative of clusters of diverse origins. Smaller dark grey boxes indicate doublets and CCs. Main clusters are indicated by Roman numerals. **B** pSTs of strains are shown and asterisks (*) mark pSTs representing more than one ST. Boxes mark clusters of pSTs with more than 99.95% similarity. All pSTs except pST79 and pST164 belong to a single CC.

The UPGMA analysis based on the concatenated sequences revealed a high genetic diversity among the analyzed strains (Figure 3). However, groups of isolates were identified by clustering of STs. These clusters contain strains with > 99% similarity. The two main clusters (marked by I and II) of the UPGMA showed a different composition in terms of geographic origin of strains. A higher proportion of South American (54%) and European STs (65%) was located in cluster I, whereas a higher proportion of Asian STs (60%) was located in cluster II. Nine of the 20 clusters (marked by boxes) largely consisted of strains originating from the same continent (Figure 3A). The CCs that were identified by goeBURST clustered together and the DLVs and TLVs were closely related in the UPGMA too (Figure 3A).

In contrast, the pSTs were grouped into one major cluster, except for pST79 and pST164 from Ecuador that were DLV and TLV, respectively (Figure 3B). The AA-UPGMA revealed no clustering depending on the geographic origin of strains. The pSTs that were more common and belonged to different STs did not originate from the same continent as indicated by the black squares. Therefore neither geographic dependency of pST affiliation nor clustering depending of origin was present.

Comparing the results obtained by UPGMA analysis of MLST and AA-MLST data, clusters on nucleotide level were not always found on peptide level (Figures 3A and B). All STs that form a CC or doublet were characterized by the same pST (CC410 and doublet ST246-ST56 were pST1; doublet ST760-ST412 was pST6).

## Discussion

### Diversity of sequence types and peptide sequence types

In our study, the diversity of *V. parahaemolyticus* strains was analyzed by different methods, including empiric analyzes, rarefaction curves, allele-based MSTs and sequence-based UPGMAs on nucleotide as well as on peptide level. The observed diversity of (p)STs, alleles, polymorphic sites, as well as *d*_*N*_*/d*_*S*_-, *D*- and $I_{A}^{S}$ -value of our strain set were similar to those obtained for the pubMLST strain collection (Tables 1, 2 and 3). This indicates that our subset is an adequate sample of the pubMLST strain collections in regard to MLST and AA-MLST properties. All applied methods revealed a high diversity in the environmental strain collections of *V. parahaemolyticus* on global as well as on local scales, as shown by others [13,15,19,23-26,39]. This was also indicated by the results obtained by rarefaction curve calculation. Rarefaction is a data re-sampling method that indicates whether the natural diversity was sampled (curve reaches the plateau) or is still rising at the end of the collection. Even the curve for the entire pubMLST database was still rising at the total sample size, indicating that some diversity of the *V. parahaemolyticus* population remains unsampled. According to the method the dataset represents a random sample taken from a closed system of a stable spectrum of types. Like Forbes and Horne suggested for *Campylobacter*, there are two possible nonexclusive explanations [40]: First, there is a closed system with a constant and stable spectrum of types but the collection schemes were not comprehensive to encompass the total ST diversity present. Second, the assumption of the closed system is invalid for the analyzed populations. Based on the available literature and our data the most appropriate interpretation for *V. parahaemolyticus* is that the present population represents an extremely large pool of strains continuously growing due to mutation and recombination [41]. For regional subpopulations strain input could occur via human activities (e.g. disposal of contaminated seafood or ships’ ballast waters) as well as migrating birds [42-45].

The majority of the identified STs was recovered only once like shown for *V. parahaemolyticus* of different sources in Thailand [24]. The high proportion of new STs can be explained by the continuously changing genotypes via recombination esp. in environmental strains [15,46] and is indicative of a poor representation of the actual diversity of *V. parahaemolyticus* by the pubMLST dataset [24].

#### Purifying selection leads to loss of diversity on peptide level

The loss of diversity on peptide level can be explained by evolutionary negative selection of non-synonymous nucleotide changes that would result in an altered amino acid composition. In the case of *V. parahaemolyticus* 95.8% of the reduction in strain diversity stemmed from the wobble bases. This is reflected by the *d*_*N*_*/d*_*S*_ value. For all loci *d*_*N*_*/d*_*S*_ was zero or close to zero, indicating purifying selection for all loci, as shown by others [15]. This is consistent with the assumption that non-synonymous substitutions lead to deleterious effects in housekeeping genes due to disrupted functions of the corresponding enzyme and even small changes (replacement of a single amino acid) may lead to a non-functional enzyme and thus may have a deleterious effect for the bacterium [28,47]. This finding is also supported by the fact that in most cases only a few different allele per locus are present and the loci are dominated by a single allele on peptide level (Additional file 1: Table S1 and Additional file 2: Table S2).

#### Distribution of sequence types and peptide sequence types

As outlined by Forbes and Horne strains of the same ST or CC are assumed to have a common ancestor, which is supposed to be more recent for strains of one ST than for strains in the same CC [40]. We hypothesize that different STs developed from a common ancestor, diversify further into a CC and result in an altered pST if sufficient genetic changes have occurred.

The global distribution of pSTs could be explained by the global dissemination of strains due to transfer of *V. parahaemolyticus* via e.g. birds or ships’ ballast waters [43,44,48]. Then the strain (of a distinct ST) would evolve locally into a distinct STs still belonging to the same pST.

Even in the different geographical subsets we could identify the common pSTs, whereas the rare pSTs were mostly recovered from a single strain set. This could be due to the local emergence of new pSTs. Similarly in the global strain set as well as the pubMLST set the rare pSTs were restricted to a single continent and the common types spread worldwide. The comparable higher diversity on pST level in Sri Lankan strains may thus be explained by the presence of established communities of *V. parahaemolyticus* that have evolved genetic changes without deleterious effects.

From Sri Lanka more STs were recovered frequently even in distinct regions, leading to the assumption that strains were distributed among farms possibly due to transmissions via different vectors, like intake seawater, feed, contaminated equipment or larvae [49,50]. Some STs were repeatedly detected at different time points. These strains seem to be well adapted to the environmental conditions at prawn farms as shown by Ellis *et al*. for *V. parahaemolyticus* in New Hampshire shellfish waters [23].

In most cases no global dissemination of environmental STs was observed. Like observed by Johnson *et al*. within different subsets, locally restricted as well as supra-regional distributed STs were found [25]. With the highest number of supra-regionally distributed STs in Sri Lankan prawn farms and the least in the NB-Seas strain set. Compared to the controlled conditions in prawn farms (e.g. constant pH and salinity) the marine and estuarine environment in the NB-Seas region is characterized by environmental changes like shifts in temperature and salinity and a plethora of different niches (e.g. open water, estuary, sediments), and may lead to the local emergence of better adapted types [51,52]. For example STs that were frequently identified within our study were either present in the North Sea or the Baltic Sea but not in both. Thus the natural subdivision of North Sea and Baltic Sea seems to represent different habitats to which different strains may be better adapted to.

Possibly the differences of ST-distribution in Sri Lankan and Ecuadorian prawn farms could be based on differing structures within shrimp farms, e.g. approx. 50% of the purchased post larvae in Sri Lankan shrimp ponds were obtained from only four vendors (one vendor supplies 24.1% of ponds), whereas in Ecuador all farms we included in our study purchased their post larvae from individual vendors ([51], unpublished data).

In single cases we were able to trace individual STs along the food chain: from seafood producing areas like Sri Lanka and Ecuador up to the retail level in Germany.

Additional analysis of the genetic diversity on smaller geographical scales (e.g. on a single farm, in a distinct bight) may help to understand if the singletons STs (or pSTs) represent locally and environmentally adapted types with a clonal structure. On the other hand low scale strain communities could also be diverse due to the introduction of new strains or genetic exchange within present types and mutational events.

Clusters of STs were identified by UPGMA that were dependent on the geographic origin and represented the local distribution of STs. Similarly, González-Escalona *et al*. observed a distinct cluster of strains isolated from patients after the consumption of raw oysters from the U.S. Pacific coast [13]. But in our data, multiple clusters per continent were identified and the distribution of STs was independent of the geographic origin (e.g. STs of all continents are scattered over the whole UPGMA tree).

On peptide level the loss of geographical clusters of pSTs in the corresponding UPGMA tree was due to the global dissemination of pSTs. Like Osorio *et al*. showed, on peptide level nearly all pSTs were grouped in one cluster [28].

By comparing the results obtained by UPGMA analysis of MLST and AA-MLST data, clusters on nucleotide level were not always found on peptide level (Figures 3A and B). But all STs that form a CC or doublet were characterized by the same pST (CC410 and doublet ST246-ST56 were pST1; doublet ST760-ST412 was pST6). This showed that both typing schemes provided different clustering results due to the decreased resolution of the AA-MLST approach, but with concordance in grouping CCs and doublets emphasizing the high degree of genetic similarity found within these groups. In the case of using a sequence based UPGMA tree no additional information was gained by application of AA-MLST analysis.

### Population structure of *V. parahaemolyticus*

The observed values of $I_{A}^{S}$ were significantly different to zero for all analyzed sets and dropped if only one isolate per ST was regarded (Table 3). These observations correspond to previous investigations and are typical for epidemic populations [13,15,23,24,27]. In these populations clones emerge from a background of recombinogetic bacteria occasionally and are able to spread [53]. In clonal populations, recombination does not occur freely and there is no random distribution of alleles in general, but recombination can occur within different subpopulations [13]. Thus our data support the postulated population structure of *V. parahaemolyticus* which follows the ‘epidemic’ model of clonal expansion [15-17,19].

#### Clonal relationships of isolates

Only 3 CCs or doublets were identified in the ‘population snapshot’. This is in agreement with the study by Turner *et al*. who also identified a low number of SLVs [27]. The CCs were either distributed in one or two continents like demonstrated before for the pandemic CC3 by González-Escalona *et al*. [13]. So far this was not shown for CCs, consisting of exclusively environmental isolates. On regional level only one triplet was identified in the Sri Lankan subset (Figure 2A). This is in concordance with Gavilan *et al.* who recorded only one CC within a geographically restricted population in Peru [29]. Thus the high degree of allelic diversity led to a decreased ability of goeBURST to identify related genotypes. Only for identical or closely related strains (SLVs to TLVs), relationships are reliable [54]. However, when strains are more distantly related, little information can be gained regarding their relationships and descent. Using pSTs instead of STs allowed an identification of strains that were closely related independent of their origin.

On pST level the ‘population snapshot’ consists of a single CC which is founded by two pSTs as shown by Theethakaew *et al*. [24]. These pSTs represent a large number of different STs of various geographic origins (pST1 corresponds to 142 STs and pST2 to 127 STs). Likely, these two pSTs represent ancestral types of *V. parahaemolyticus*. Other pSTs might have arisen from these ancestral types via genetic drift associated with mutational or other genetic changes [28]. A similar result has been observed by Osorio *et al.* who applied a peptide based MLST-scheme to *Brachyspira hyodysenteriae*, to deduce putative ancestral relationships between different strains [28].

In context of all pubMLST isolates the formation of the new CC412 was observed. This CC was founded by the environmental ST412 and harbors on SLV to TLV level potentially pathogenic environmental as well as clinical strains. This emphasizes the close genetic relatedness of environmental and infectious STs as already observed by Ellis *et al*. [23]. Due to the presence of these STs in the same habitat, virulence genes can be exchanged via recombination or transfer of mobile elements [55]. However, the membership of a ST in a CC with pathogenic strains allows no prediction of the pathogenic properties of this ST [17].

### Comparison of MST and UPGMA

The geographic dependency found in UPGMAs but not in MSTs could be explained by the different approaches of sequence-based versus allelic profile-based comparison. Sequences with fewer differences are grouped close together in the UPGMA whereas in MSTs all sequences which differ in at least one nucleotide have the same distance to each other. Thus the UPGMA seems to be more suitable for showing geographical relationships between strains of highly diverse populations.

The CCs identified by goeBURST were grouped together also in UPGMA analysis. Similarly Yan *et al*. observed the grouping of CCs identified by eBURST in high monophyletic clades of UPGMA analysis [15].

## Conclusions

The generated data reveal a high genetic diversity for all *V. parahaemolyticus* strain subsets analyzed, with a high proportion of new alleles and STs discovered, typical for environmental strain collections. Clusters of strains on nucleotide level contained mainly strains originating from one continent, but no exclusive clusters for the distinct continents were identified. STs and pSTs were either supra-regionally distributed or exclusively present in one region. Using AA-MLST instead of MLST in the goeBURST analysis allowed reliable identification of closely related strains (pSTs were SLVs), independent of their geographic origin. In contrast the application of MLST is more useful to recognize relationships in an epidemiological context by creating distinct CCs.

In general pubMLST database reflects only the diversity of so far analyzed strains, and may not represent the natural diversity of the *V. parahaemolyticus* population as also indicated by our rarefaction analysis. Further analysis of strains of diverse origins may help to complete the database and to keep pace with new evolving genotypes.

### Availability of supporting data

The data sets and additional figures supporting the results of this article are included in Additional files 1, 2, 3, 4 and 5.

## Abbreviations

V: *Vibrio*; ST: Sequence type; pST: Peptide sequence type; CC: Clonal complex; SLV: Single locus variant; DLV: Double locus variant; TLV: Triple locus variant; AA-MLST: Amino acid-MLST; tdh: Thermostable direct hemolysin gene; trh: *tdh*-related hemolysin gene; GS-PCR: Group specific-PCR; NB-Seas: North Sea, Baltic Sea, Kattegat and Skagerrak; APW: Alkaline peptone water; MST: Minimum spanning tree; UPGMA: Unweighted pair group method with arithmetic mean; SNP: Single nucleotide polymorphism; $I_{A}^{S}$: Index of Association; D: Simpsons index of diversity.

## Competing interests

The authors declare that they have no competing interests.

## Author’s contributions

SU did the experimental design, performed the experiments, analyzed the data and drafted the manuscript. TA and SH participated in study design, data analysis and drafting the manuscript. GG participated in selection of strains and drafting the manuscript. MK, LS and UM took part in preparing and performing the experiments. All authors have read and approved the manuscript.

## Supplementary Material

Additional file 1: Table S1 Characteristics and allelic profiles of *V. parahaemolyticus* strains included within this study. Click here for file

Additional file 2: Tables S2 AA-MLST profiles and properties of each allele on peptide level (numbers, sequences and frequencies). Click here for file

Additional file 3: Figure S1 Population snapshot based on MLST profiles of pubMLST dataset. Coloring depends on geographical origin of isolates: Asia (red), South America (light green), North America (dark green), Africa (yellow) and Europe (blue). Size of circles represents number of isolates with the corresponding ST. STs that differ in one allele are connected via black lines. Click here for file

Additional file 4: Figure S2 FullMST based on MLST profiles of pubMLST dataset. Coloring depends on geographical origin of isolates: Asia (red), South America (light green), North America (dark green), Africa (yellow) and Europe (blue). Size of circles represents number of isolates with the corresponding ST. All connections were drawn. SLVs are connected via black, DLVs via dark grey, TLVs via grey and all connection with a higher level via light grey lines. Click here for file

Additional file 5: Figure S3 FullMST based on AA-MLST profiles of pubMLST dataset. Coloring depends on geographical origin of isolates: Asia (red), South America (light green), North America (dark green), Africa (yellow) and Europe (blue). Size of circles represents number of isolates with the corresponding pST. All connections were drawn. SLVs are connected via black, DLVs via dark grey and TLVs via grey lines. Click here for file
