# Supplementary material for: Population analysis of Vibrio parahaemolyticus originating from different geographical regions demonstrates a high genetic diversity
Source: BMC Microbiol. 2014 Mar 8;14:59. doi: 10.1186/1471-2180-14-59 (PMC4015679; doi:10.1186/1471-2180-14-59)
Supplement: Additional file 1: Table S1 — Characteristics and allelic profiles of V. parahaemolyticus strains included within this study. [file 1471-2180-14-59-S1.pdf]

Table S1: Characteristics and allelic profiles of *V. parahaemolyticus* strains included within this study.

	Origin			Allelic profile[A]			
id[B]	Country, Region	Year	Source[C]	Nucleotide	Peptide	ST	pST
816	Sri Lanka, Chillaw	2010	Prawn	11-75-64-50-26-7-50	2-1-1-1-1-1-1	**409**	2
817	Sri Lanka, Chillaw	2010	Prawn	11-75-64-50-26-7-50	2-1-1-1-1-1-1	**409**	2
794	Sri Lanka, Chillaw	2010	Prawn	**166-211-162-183**-50-3-57	1-1-1-1-1-2-1	**394**	3
818	Sri Lanka, Chillaw	2010	Prawn	11-75-64-50-26-7-50	2-1-1-1-1-1-1	**409**	2
795	Sri Lanka, Chillaw	2010	Prawn	**169-104**-151-73-26-46-94	1-1-1-1-1-3-1	**395**	4
796	Sri Lanka, Chillaw	2010	Prawn	9-**213-165-185**-2-46-1	2-1-1-1-2-3-1	**396**	20
791	Sri Lanka, Chillaw	2010	Prawn	9-**213-165-185**-2-46-1	2-1-1-1-2-3-1	**396**	20
797	Sri Lanka, Chillaw	2010	Prawn	26-**218**-124-94-23-119-13	1-1-1-1-1-1-1	**397**	1
819	Sri Lanka, Chillaw	2010	Prawn	26-**218**-124-94-23-119-13	1-1-1-1-1-1-1	**397**	1
792	Sri Lanka, Chillaw	2010	Prawn	26-**218**-124-94-23-119-13	1-1-1-1-1-1-1	**397**	1
798	Sri Lanka, Chillaw	2010	Prawn	**165**-22-70-**177**-39-11-**115**	1-1-1-**9**-1-1	**398**	**85**
820	Sri Lanka, Puttalam	2011	Prawn	153-191-70-19-23-8-1	1-1-1-1-1-1-1	355	1
821	Sri Lanka, Puttalam	2011	Prawn	**166-211-162-183**-50-3-57	1-1-1-1-1-2-1	**394**	3
822	Sri Lanka, Puttalam	2011	Prawn	**166-211-162-183**-50-3-57	1-1-1-1-1-2-1	**394**	3
799	Sri Lanka, Puttalam	2011	Prawn	**166-211-162-183**-50-3-57	1-1-1-1-1-2-1	**394**	3
823	Sri Lanka, Puttalam	2011	Prawn	153-58-70-19-23-8-1	1-1-1-1-1-1-1	**410**	1
914	Sri Lanka, Puttalam	2011	Prawn	**166-211-162-183**-50-3-57	1-1-1-1-1-2-1	**394**	3
800	Sri Lanka, Puttalam	2011	Prawn	153-**216**-70-19-23-8-1	1-1-1-1-1-1-1	**399**	1
801	Sri Lanka, Puttalam	2011	Prawn	12-**208-159**-133-28-128-86	2-1-1-1-1-1-1	**400**	2
824	Sri Lanka, Puttalam	2011	Prawn	**166-211-162-183**-50-3-57	1-1-1-1-1-2-1	**394**	3
802	Sri Lanka, Puttalam	2011	Prawn	112-**217-145-178-111-160-116**	1-1-1-1-**13**-1-1	**401**	**69**
825	Sri Lanka, Puttalam	2011	Prawn	26-**218**-124-94-23-119-13	1-1-1-1-1-1-1	**397**	1
803	Sri Lanka, Puttalam	2011	Prawn	80-25-**160-179**-26-10-**117**	1-1-1-1-1-1-**20**	**402**	**39**
826	Sri Lanka, Puttalam	2011	Prawn	26-**218**-124-94-23-119-13	1-1-1-1-1-1-1	**397**	1
804	Sri Lanka, Puttalam	2011	Prawn	**167**-25-25-91-18-57	2-1-1-1-1-1-1	**403**	2
827	Sri Lanka, Puttalam	2011	Prawn	**166-211-162-183**-50-3-57	1-1-1-1-1-2-1	**394**	3
915	Sri Lanka, Puttalam	2011	Prawn	**166-211-162-183**-50-3-57	1-1-1-1-1-2-1	**394**	3
828	Sri Lanka, Madurankuliya	2011	Prawn	**169-104**-151-73-26-46-94	1-1-1-1-1-3-1	**395**	4
916	Sri Lanka, Madurankuliya	2011	Prawn	26-**218**-124-94-23-119-13	1-1-1-1-1-1-1	**397**	1
917	Sri Lanka, Madurankuliya	2011	Prawn	**169-104**-151-73-26-46-94	1-1-1-1-1-3-1	**395**	4
793	Sri Lanka, Madurankuliya	2011	Prawn	112-**217-145-178-111-160-116**	1-1-1-1-**13**-1-1	**401**	**69**
829	Sri Lanka, Puttalam	2011	Prawn	**169-104**-151-73-26-46-94	1-1-1-1-1-3-1	**395**	4
918	Sri Lanka, Madurankuliya	2011	Prawn	**166-211-162-183**-50-3-57	1-1-1-1-1-2-1	**394**	3
919	Sri Lanka, Madurankuliya	2011	Prawn	26-**220-168**-159-11-**168-122**	1-1-1-1-1-1-1	**419**	1
920	Sri Lanka, Madurankuliya	2011	Prawn	**169-104**-151-73-26-46-94	1-1-1-1-1-3-1	**395**	4
921	Sri Lanka, Madurankuliya	2011	Prawn	153-191-70-19-23-8-1	1-1-1-1-1-1-1	355	1
922	Sri Lanka, Puttalam	2011	Prawn	131-**221-163**-150-**114-163**-26	1-1-1-1-1-1-1	**420**	1
830	Sri Lanka, Puttalam	2011	Prawn	26-**218**-124-94-23-119-13	1-1-1-1-1-1-1	**397**	1
923	Sri Lanka, Puttalam	2011	Prawn	153-191-70-19-23-8-1	1-1-1-1-1-1-1	355	1
924	Sri Lanka, Madurankuliya	2011	Prawn	153-191-70-19-23-8-1	1-1-1-1-1-1-1	355	1
925	Sri Lanka, Puttalam	2011	Prawn	11-117-123-**184-159-169**-57	2-1-1-1-**34**-1-1	**673**	**136**
926	Sri Lanka, Madurankuliya	2011	Prawn	**169-104**-151-73-26-46-94	1-1-1-1-1-3-1	**395**	4
927	Sri Lanka, Madurankuliya	2011	Prawn	**169-104**-151-73-26-46-94	1-1-1-1-1-3-1	**395**	4
808	Pacific	2010	Mussel	5-**215**-19-120-46-110-23	2-1-1-1-1-2-1	**404**	5
928	Thailand	2008	Prawn	5-84-115-74-63-159-84	2-1-1-1-1-1-1	392	2
929	Bangladesh	2008	Prawn	157-124-135-73-19-**170-123**	1-1-1-1-1-1-1	**422**	1
930	Vietnam	2008	Prawn	51-4-77-67-60-8-24	2-1-1-1-1-1-1	**423**	2
931	India	2008	Prawn	**170-224**-75-139-**117**-18-**124**	1-1-1-1-1-1-1	**424**	1
1059	Indian Ocean	2008	Prawn	**166-211-162-183**-50-3-57	1-1-1-1-1-2-1	**394**	3
932	Bangladesh	2008	Prawn	121-50-**169**-91-10-73-73	2-1-1-1-1-1-1	**425**	2
831	Thailand	2008	Prawn	55-15-31-55-18-58-46	1-1-1-1-1-5-1	114	10
933	Honduras	2008	Prawn	158-**223**-75-12-26-**171-125**	2-1-1-1-1-1-**21**	**426**	**100**
934	Honduras	2008	Prawn	33-87-24-5-10-5-1	1-1-1-1-1-1-1	246	1
935	Vietnam	2009	Prawn	**171-222**-113-126-4-62-23	2-1-1-1-1-1-1	**428**	2
832	Thailand	2009	Prawn	119-152-135-29-23-11-61	2-1-1-1-1-1-1	**391**	2
805	Bangladesh	2010	Prawn	33-22-24-5-10-5-1	1-1-1-1-1-1-1	56	1
833	Vietnam	2010	Prawn	2-113-72-94-26-83-23	1-1-1-1-1-2-1	**411**	3
810	Bangladesh	2011	Prawn	31-4-60-**180**-31-62-**120**	1-1-1-1-1-1-2	**405**	6
812	Thailand	2011	Prawn	**168-210-161-182**-61-**162**-38	2-1-1-1-1-1-1	**406**	2
1046	Netherlands, North Sea	2011	Prawn	**221-298**-31-**226**-26-171-37	2-1-1-1-1-1-1	**545**	2
1047	Denmark	2011	Prawn	119-151-**197**-79-4-**205**-105	2-1-1-1-1-1-1	**537**	2

id[B]	Origin			Allelic profile[A]		ST	pST
	Country, Region	Year	Source[C]	Nucleotide	Peptide		
1048	Denmark	2011	Prawn	119-151-**197**-79-4-**205**-105	2-1-1-1-1-1-1	**537**	2
1049	Denmark	2011	Prawn	119-151-**168**-201-4-**205**-105	2-1-1-1-1-1-1	**538**	2
813	Vietnam	2011	Prawn	102-**212**-91-**182**-61-**162**-38	2-1-1-1-1-1-1	**407**	2
814	China	2011	Prawn	111-**188**-**164**-149-**115**-**164**-**118**	1-1-1-1-1-**36**-1	**408**	**60**
834	Vietnam	2011	Prawn	109-136-114-121-83-107-83	2-1-1-1-1-2-1	233	5
835	Ecuador	2011	Prawn	142-29-10-7-14-24-20	1-1-1-1-1-1-2	**412**	6
836	Bangladesh	2011	Prawn	35-154-25-50-73-35-23	1-1-1-1-1-4-1	253	11
837	Germany, Baltic Sea	2011	Water	3-2-82-50-4-78-66	2-1-1-1-1-1-1	121	2
838	Germany, Baltic Sea	2011	Seaweed	35-43-38-21-31-35-37	1-1-1-1-1-4-1	79	11
839	Germany, Baltic Sea	2011	Sand	3-2-82-50-4-78-66	2-1-1-1-1-1-1	121	2
840	Germany, Baltic Sea	2011	Water	3-2-82-50-4-78-66	2-1-1-1-1-1-1	121	2
841	Germany, Baltic Sea	2011	Water	3-2-82-50-4-78-66	2-1-1-1-1-1-1	121	2
1050	Ecuador	2011	Prawn	31-**299**-**198**-19-26-**206**-18	1-1-1-1-1-2-1	**539**	3
1051	Ecuador	2011	Prawn	42-**223**-31-12-26-**171**-57	2-1-1-1-1-1-1	**540**	2
1052	NE-Atlantic	2011	Prawn	17-**296**-13-**224**-95-31-26	1-1-1-1-1-1-1	**541**	1
1053	Netherlands, North Sea	2011	Prawn	175-16-**195**-**187**-31-**165**-17	1-1-1-1-1-**21**-1	**542**	**54**
1054	Ecuador, Machala	2012	Prawn	188-106-**199**-**225**-2-3-1	2-1-1-1-2-2-1	**543**	**123**
1055	Ecuador, Machala	2012	Prawn	42-**297**-**196**-19-**137**-**204**-57	2-1-1-1-**15**-1-1	**544**	**129**
1056	Ecuador, Machala	2012	Prawn	42-**297**-**196**-19-**137**-**204**-57	2-1-1-1-**15**-1-1	**544**	**129**
1137	Ecuador, Machala	2012	Prawn	111-7-**232**-12-**166**-**257**-**182**	1-1-1-1-**35**-3-**24**	**718**	**79**
1057	Ecuador, Machala	2012	Prawn	42-**223**-31-12-26-**171**-57	2-1-1-1-1-1-1	**540**	2
1138	Ecuador, Machala	2012	Prawn	58-**332**-10-8-28-33-**183**	1-1-1-1-1-1-1	**719**	1
1139	Ecuador, Machala	2012	Prawn	**236**-40-38-209-28-190-94	**15**-1-1-3-1-18-1	**720**	**164**
1140	Ecuador, Machala	2012	Prawn	60-143-**233**-29-47-78-17	1-1-1-1-1-1-1	**721**	1
1141	Ecuador, Machala	2012	Prawn	26-16-**234**-7-18-32-7	1-1-1-1-1-1-1	**722**	1
1142	Ecuador, Balao	2012	Prawn	232-141-3-54-160-**258**-90	2-1-1-1-1-1-6	**723**	**95**
1143	Ecuador, Balao	2012	Prawn	42-**333**-74-**288**-24-171-13	2-1-1-1-1-1-1	**724**	2
1207	Ecuador, Machala	2012	Prawn	28-84-217-**290**-28-**260**-**184**	1-1-1-1-1-1-**25**	**756**	**41**
1145	Ecuador, Machala	2012	Prawn	26-16-**234**-7-18-32-7	1-1-1-1-1-1-1	**722**	1
1146	Ecuador, Machala	2012	Prawn	26-16-**234**-7-18-32-7	1-1-1-1-1-1-1	**722**	1
1147	Ecuador, Machala	2012	Prawn	47-**335**-61-**289**-114-78-26	1-1-1-1-1-1-1	**725**	1
1149	Ecuador, Machala	2012	Prawn	**170**-**224**-75-139-**117**-18-**124**	1-1-1-1-1-1-1	**424**	1
1148	Ecuador, Machala	2012	Prawn	31-**334**-3-55-4-**259**-23	1-1-1-1-1-3-1	**755**	4
1150	Ecuador, Guayaquil	2012	Prawn	26-16-**234**-7-18-32-7	1-1-1-1-1-1-1	**722**	1
1151	Ecuador, Guayaquil	2012	Prawn	**237**-165-98-**291**-**167**-11-77	1-1-1-1-1-1-1	**726**	1
1152	Ecuador, Guayaquil	2012	Prawn	31-115-200-12-**168**-91-68	1-1-1-1-1-12-1	**727**	17
1153	Ecuador, Guayaquil	2012	Prawn	31-115-200-12-**168**-91-68	1-1-1-1-1-12-1	**727**	17
1154	Ecuador, Guayaquil	2012	Prawn	28-84-217-**290**-28-**260**-**184**	1-1-1-1-1-1-**25**	**756**	**41**
1155	Ecuador, Guayaquil	2012	Prawn	5-25-19-61-4-11-24	2-1-1-1-1-1-1	430	2
1156	Ecuador, Guayaquil	2012	Prawn	25-322-13-282-33-32-2	1-1-1-1-1-1-1	711	1
1157	Ecuador, Guayaquil	2012	Prawn	196-84-80-124-128-158-12	2-1-1-1-1-14-1	**757**	**29**
1158	Ecuador, Guayaquil	2012	Prawn	26-16-**234**-7-18-32-7	1-1-1-1-1-1-1	**722**	1
1159	Ecuador, Guayaquil	2012	Prawn	232-56-3-23-56-11-36	2-1-1-1-4-1-1	**728**	14
1211	Germany, North Sea	2011	Water	**238**-106-75-123-30-11-46	**9**-1-1-1-1-1-1	**758**	**158**
1212	Germany, North Sea	2011	Water	**239**-16-13-8-49-**261**-23	4-1-1-1-1-1-1	**759**	**151**
1213	Germany, North Sea	2011	Water	142-16-10-7-14-24-20	1-1-1-1-1-1-2	**760**	6
1214	Germany, Baltic Sea	2011	Water	4-13-11-91-18-9-23	2-1-1-1-1-7-1	481	19
1215	Germany, Baltic Sea	2011	Water	4-13-11-91-18-9-23	2-1-1-1-1-7-1	481	19
1216	Germany, Baltic Sea	2011	Water	47-52-19-287-24-43-37	1-1-1-1-1-1-1	**761**	1
1217	Germany, Baltic Sea	2011	Water	**240**-**337**-**240**-19-**169**-**262**-**185**	4-1-1-1-1-6-1	**762**	**152**
1218	Germany, Baltic Sea	2011	Water	33-280-**239**-**292**-23-**263**-83	1-1-1-1-1-1-1	**763**	1
1219	Germany, North Sea	2011	Water	142-16-10-7-14-24-20	1-1-1-1-1-1-2	**760**	6
1220	Germany, North Sea	2011	Water	142-16-10-7-14-24-20	1-1-1-1-1-1-2	**760**	6
1221	Germany, North Sea	2011	Water	27-327-13-23-33-32-2	1-1-1-1-1-1-1	**764**	1
1222	Denmark, North Sea	2011	Phytopl.	27-327-13-23-33-32-2	1-1-1-1-1-1-1	**764**	1
1223	Denmark, Skagerrak	2011	Water	61-117-35-19-26-92-**186**	1-1-1-1-1-13-1	**765**	**48**
1224	Denmark, Kattegat	2011	Water	26-330-223-**293**-28-**264**-104	1-1-1-1-1-2-1	**774**	3
1225	Denmark, Kattegat	2011	Zoopl.	22-**338**-**241**-205-4-**265**-86	2-1-1-1-1-**41**-1	**766**	**119**
1226	Denmark, Kattegat	2011	Phytopl.	111-239-205-195-28-46-23	1-1-1-1-1-3-1	**767**	4
1227	Germany, Baltic Sea	2011	Water	29-**339**-75-123-30-18-46	1-1-1-1-1-1-1	**768**	1
1228	Germany, Baltic Sea	2011	Zoopl.	17-16-13-7-24-16-20	1-1-1-1-1-1-2	28	6
1229	Germany, Baltic Sea	2011	Sediment	196-84-80-**294**-**170**-158-12	2-1-1-1-1-14-1	**769**	**29**
1230	Germany, Baltic Sea	2011	Water	4-13-11-91-18-9-23	2-1-1-1-1-7-1	481	19
1231	Germany, Baltic Sea	2011	Water	97-287-150-243-142-229-**187**	2-1-1-1-1-1-1	**770**	2

id[B]	Origin: Country, Region	Year	Source[C]	Allelic profile[A]: Nucleotide	Peptide	ST	pST
1232	Germany, Baltic Sea	2011	Phytopl.	20-**296**-3-187-26-6-2	1-1-1-1-1-5-1	**771**	10
1233	Germany, Baltic Sea	2011	Water	47-58-53-19-50-37-26	1-1-1-1-1-2-1	162	3
1234	Germany, Baltic Sea	2011	Water	17-**340**-6-**295**-94-23-7	1-1-1-1-1-1-1	**773**	1
1235	Germany, Baltic Sea	2011	Water	4-13-11-91-18-9-23	2-1-1-1-1-7-1	481	19
1236	Germany, North Sea	2011	Water	**238**-106-75-123-30-11-46	**9**-1-1-1-1-1-1	**758**	**158**

Numbers in bold in the allelic profile, ST and pST correspond to sequences or types newly described.

[A] Allelic profile on basis of nucleotide and peptide sequences. Order of fragments: *dna*E, *gyr*B, *rec*A, *dtd*S, *pnt*A, *pyr*C, *tna*A and p_dnaE, p_gyrB, p_recA, p_dtdS, p_pntA, p_pyrC, p_tnaA.

[B] id of the isolate as assigned by the pubMLST database after submission

[C] Phytopl. = Phytoplankton, Zoopl. = Zooplankton
