# Supplementary figures and images for: Population analysis of Vibrio parahaemolyticus originating from different geographical regions demonstrates a high genetic diversity
Source: BMC Microbiol. 2014 Mar 8;14:59. doi: 10.1186/1471-2180-14-59 (PMC4015679; doi:10.1186/1471-2180-14-59)

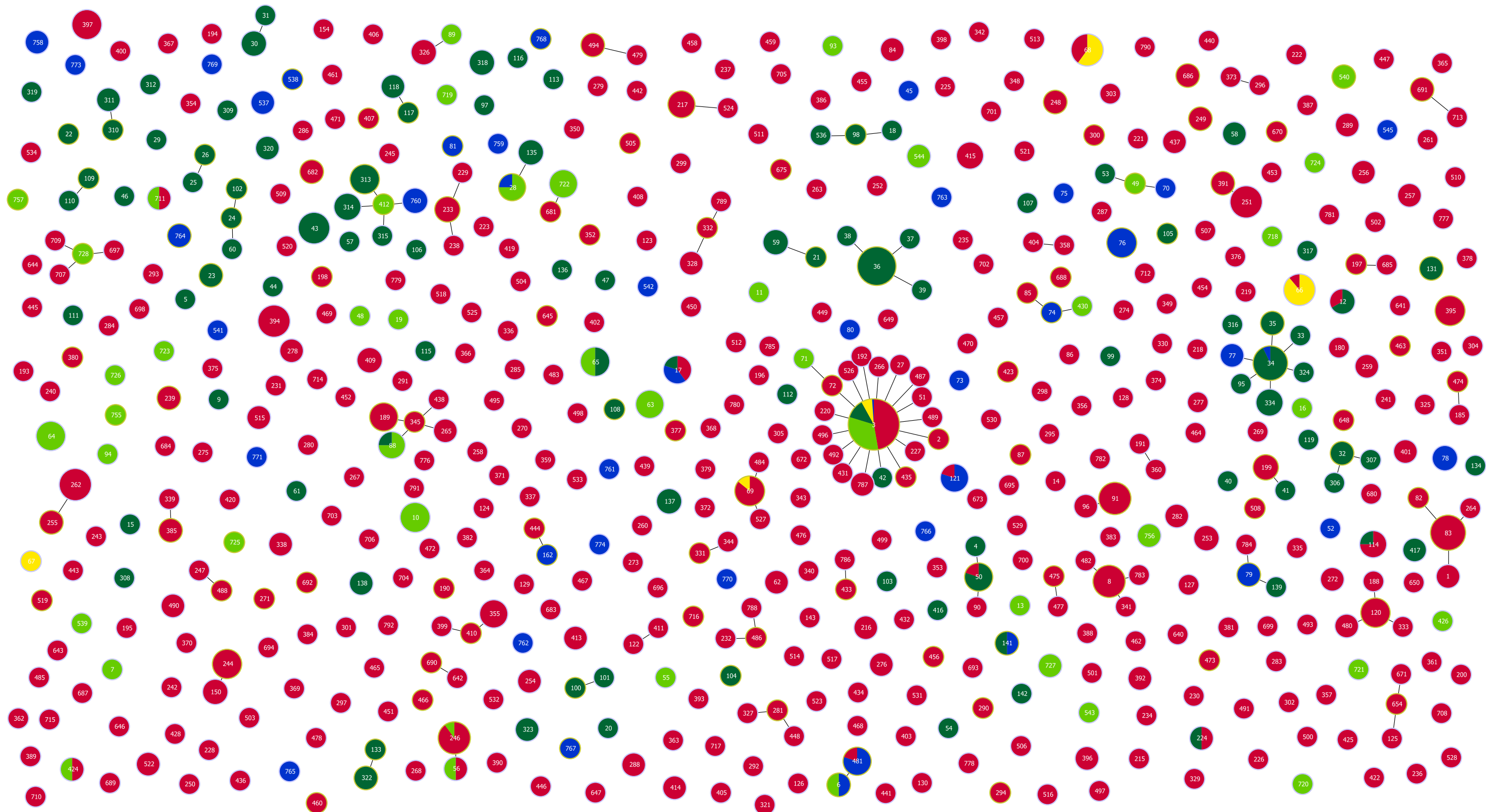

Supplement: Additional file 3: Figure S1 — Population snapshot based on MLST profiles of pubMLST dataset. Coloring depends on geographical origin of isolates: Asia (red), South America (light green), North America (dark green), Africa (yellow) and Europe (blue). Size of circles represents number of isolates with the corresponding ST. STs that differ in one allele are connected via black lines. [file 1471-2180-14-59-S3.pdf]

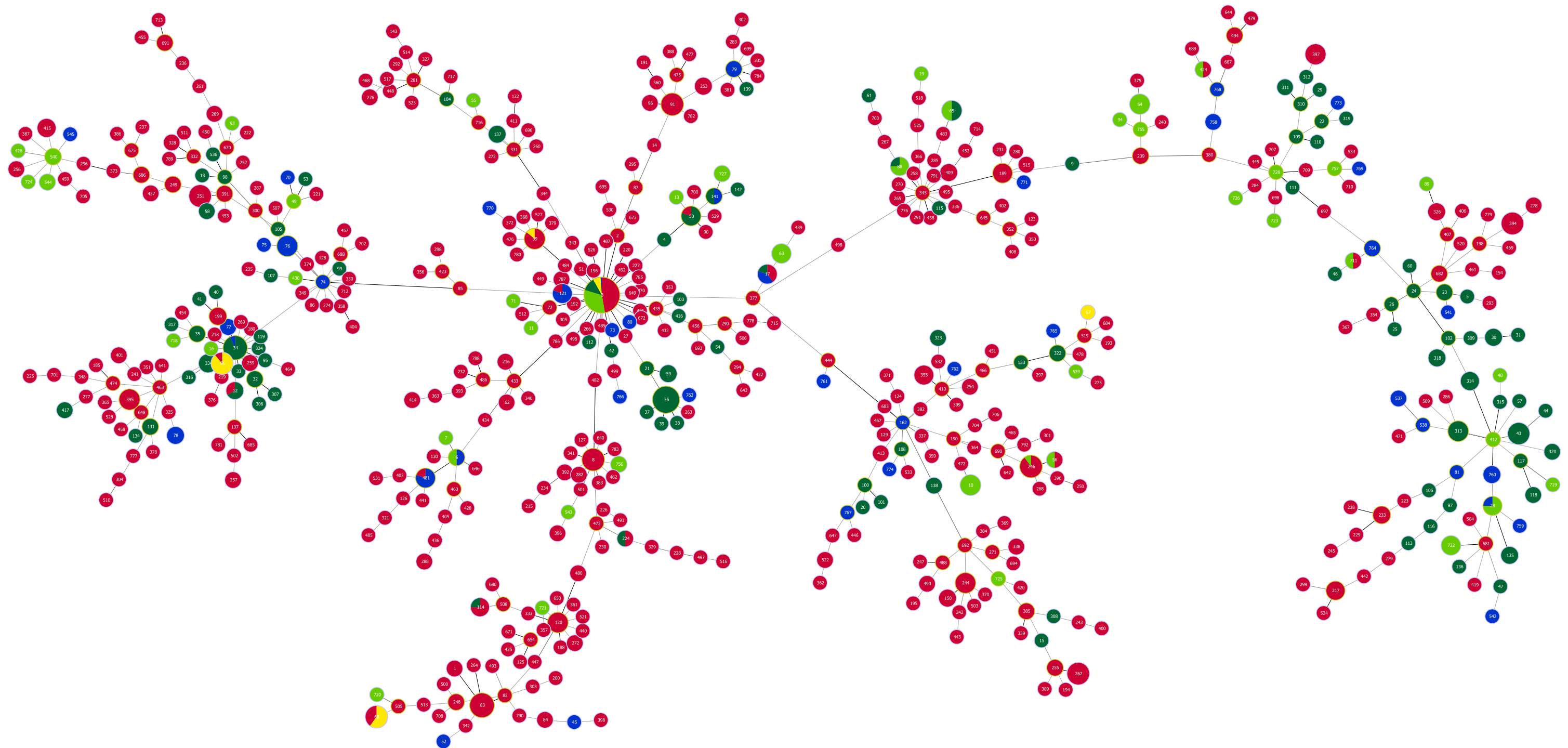

Supplement: Additional file 4: Figure S2 — FullMST based on MLST profiles of pubMLST dataset. Coloring depends on geographical origin of isolates: Asia (red), South America (light green), North America (dark green), Africa (yellow) and Europe (blue). Size of circles represents number of isolates with the corresponding ST. All connections were drawn. SLVs are connected via black, DLVs via dark grey, TLVs via grey and all connection with a higher level via light grey lines. [file 1471-2180-14-59-S4.pdf]

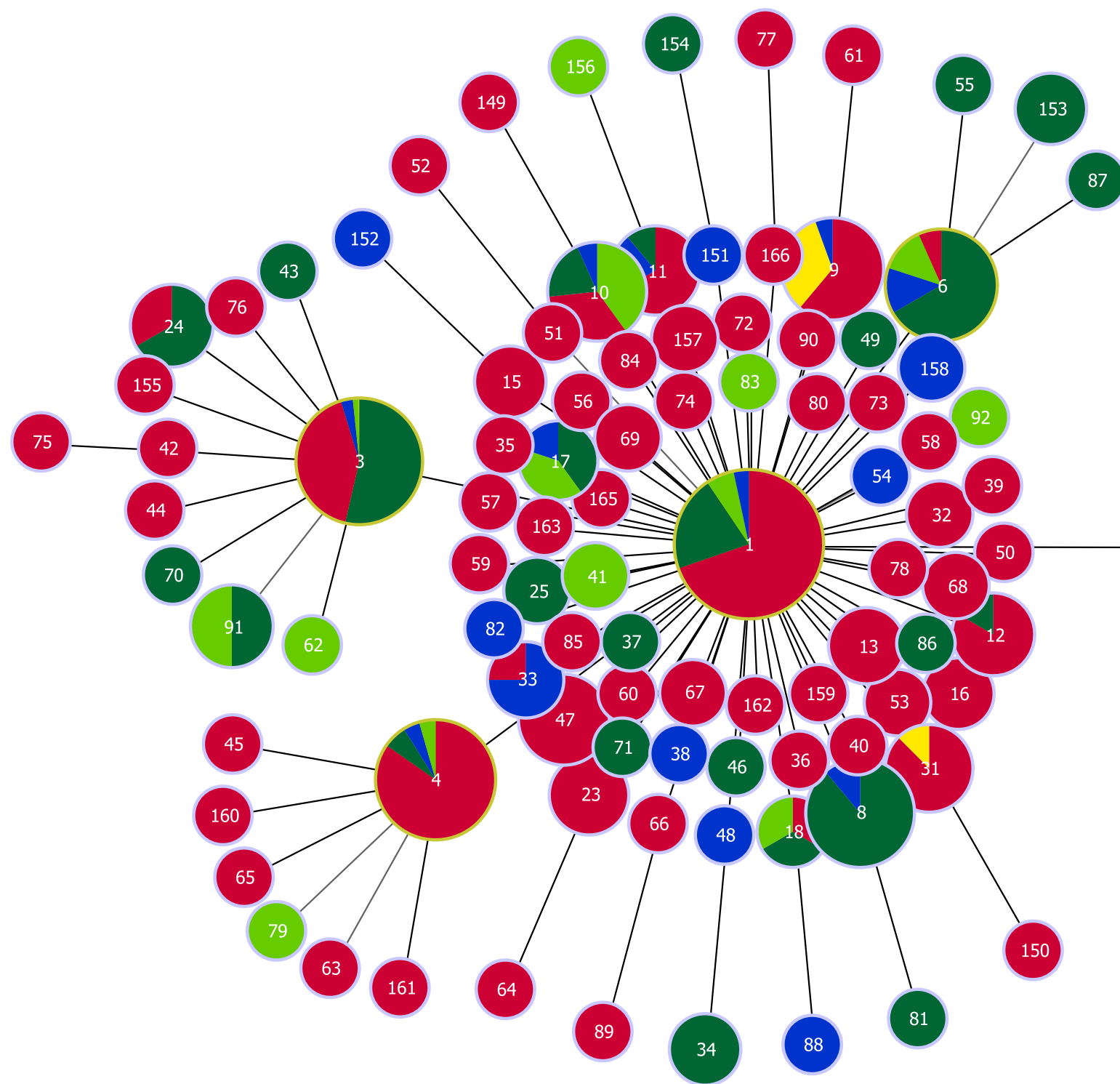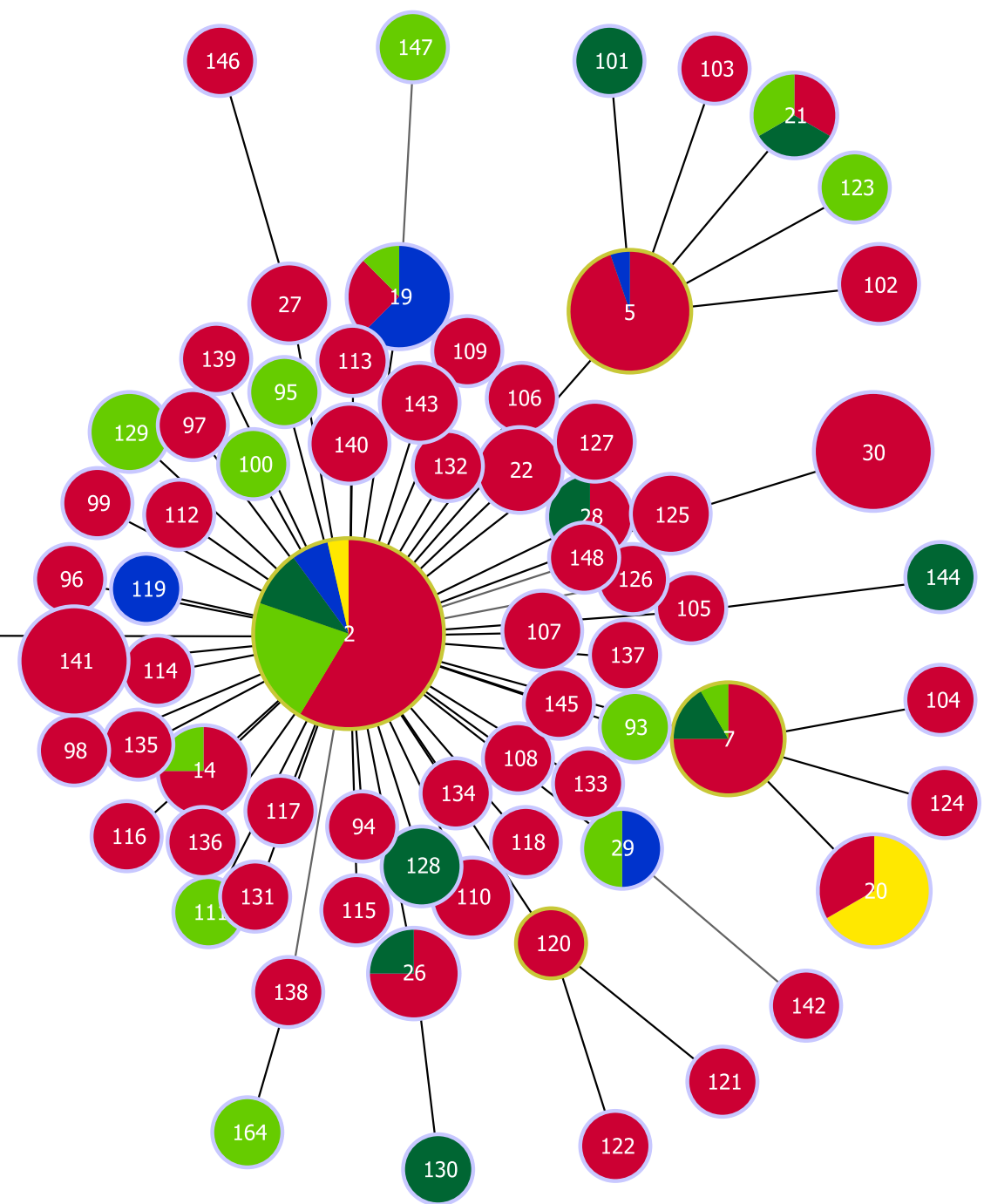

Supplement: Additional file 5: Figure S3 — FullMST based on AA-MLST profiles of pubMLST dataset. Coloring depends on geographical origin of isolates: Asia (red), South America (light green), North America (dark green), Africa (yellow) and Europe (blue). Size of circles represents number of isolates with the corresponding pST. All connections were drawn. SLVs are connected via black, DLVs via dark grey and TLVs via grey lines. [file 1471-2180-14-59-S5.pdf]
